# Clinical Profile and Outcome of Pediatric Mitochondrial Myopathy in China

**DOI:** 10.3389/fneur.2020.01000

**Published:** 2020-09-08

**Authors:** Chaoping Hu, Xihua Li, Lei Zhao, Yiyun Shi, Shuizhen Zhou, Yi Wang

**Affiliations:** Neurology Department, Children's Hospital of Fudan University, Shanghai, China

**Keywords:** mitochondrial myopathy, gene mutation, treatment, cytochrome-c oxidase, pathology

## Abstract

**Introduction:** Mitochondrial myopathy in children has notable clinical and genetic heterogeneity, but detailed data is lacking.

**Patients and Methods:** In this study, we retrospectively reviewed the clinical presentation, laboratory investigation, genetic and histopathological characteristics, and follow-ups of 21 pediatric mitochondrial myopathy cases from China.

**Results:** Twenty-four patients suspected with mitochondrial myopathy were enrolled initially and 21 were genetically identified. Fourteen patients were found to harbor mitochondrial DNA point mutations (14/21, 66.7%), including m.3243A>G (9/15, 60%), m.3303C>T (2/15, 13.3%), m.3302A>G (1/15, 6.7%), m.3250T>C (1/15, 6.7%), m.3251A>G (1/15, 6.7%), of whom 12 patients presented with progressive proximal mitochondrial myopathy (12/14, 85.7%). Three patients revealed large-scale deletion in blood or muscle tissue (3/21, 14.3%), presenting with Kearns-Sayer syndrome (1/3, 33.3%) or chronic progressive external ophthalmoplegia (2/3, 66.7%). Four patients were found to harbor pathogenic nuclear gene variants (4/21, 19.0%), including five variants in *TK2* gene and two variants in *SURF1* gene. During the follow-ups up to 7 years, 10 patients developed cardiomyopathy (10/21, 47.6%), 13 patients occurred at least once hypercapnic respiratory failure (13/21, 61.9%), six experienced recurrent respiratory failure and intubation (6/21, 28.6%), eight patients failed to survive (8/21, 38.1%). With nocturnal non-invasive ventilation of BiPAP, three patients recovered from respiratory failure, and led a relative stable and functional life (3/21, 14.3%).

**Conclusion:** Mitochondrial myopathy in children has great clinical, pathological, and genetical heterogeneity. Progressive proximal myopathy is most prevalent. Mitochondrial DNA point mutations are most common. And respiratory failure is a critical risk factor of poor prognosis.

## Introduction

Mitochondrial myopathies (MMs), are an important group of muscle conditions characterized by isolated or predominant skeletal muscle involvement, caused primarily by the impairment of oxidative phosphorylation (OXPHOS) due to mutations of nuclear or mitochondrial DNA (1). Currently there are no available effective or disease-modifying treatments for majority of patients with mitochondrial myopathies (1). Myopathy is one of the most common manifestations of adult-onset mitochondrial disorders due to the high cellular energy demand of skeletal muscle. However, there are rare reports about the clinical and genetic characteristics, natural history, and prognosis of MMs in children.

In this study, we retrospectively reviewed the clinical presentation, pathological feature, genetic characteristic, and follow up of a cohort of mitochondrial myopathy in children from China, and preliminarily analyzed the risk factors and treatments correlated with the prognosis.

## Patients and Methods

Twenty-four patients suspected with mitochondrial myopathy were enrolled, from the Department of Neurology, Children's Hospital of Fudan University between March 2011 and August 2019. The clinical presentation, laboratory data, genetic results, pathological findings, and follow up information were collected and reviewed. All patients were interviewed and examined by at least two neurologists.

Ethical approval for the study was obtained from the health authority ethical committee of Children's Hospital of Fudan University. All tissue samples were collected after obtaining written consent from parents of each patient in compliance with the Declaration of Helsinki.

### DNA Isolation, Molecular Test, and Analysis

All 24 patients received genetic tests, of which mtDNA analysis of blood and muscle tissues were performed by Jiajian medical company in Guangzhou while nDNA assays (NGS) of blood samples were performed at the Translational Research Center of Children's Hospital of Fudan University.

Between 2011 and 2013, hot spots of mtDNA mutations(3243A>G, 8344A>G, 8993T>G/C, 1555A>G, 11778G>A) were tested in 4 patients by using HPLC (high efficiency liquid chromatography). After 2014, next generation sequencing of whole mtDNA was conducted in 20 patients. Mitochondrial DNA was enriched by a single amplicon using long-range PCR of the entire mitochondrial genome, followed by analysis on an MPS deploying Illumina HiSeq 2000 system (Illumina, San Diego, CA) (2, 3). After mapping to the last Cambridge edition of the human mitochondrial genome (rCRS NC_012920) to filter variants with a certain allele frequency, an in-house database of ~20,000 mitochondrial genomes was employed for analysis. This comprehensive approach simultaneously identifies pathogenic mtDNA point mutations with quantification of the degree of heteroplasmy as low as 1.3%, with large deletions with precise breakpoints mapped and the degree of deletion heteroplasmy estimated. Targeted sequence analysis of mtDNA variants in matrilineal family members was also performed in some cases to assess pathogenicity.

Between 2011 and 2017, four patients received neuromuscular gene panel analysis, and after 2018 three patients conducted whole exon sequencing. Nuclear DNA was extracted from 2-mL aliquots of whole-blood leukocytes obtained from patients. NGS of nDNA was performed using genomic DNA from each patient on a HiSeq 2000 platform (Illumina). After mapping to the reference human genome (UCSC hg 19), sequencing data were sorted and merged, with duplicate reads removed from BAM files with SAM tools version 0.1.16. Sanger sequencing was performed to validate the variations and determine their parental origin. Quantitative analysis of mtDNA content was conducted in muscle samples of three patients with *TK2* mutations.

The variant was classified as “pathogenic” or “likely pathogenic” according to the variant interpretation guidelines of the American College of Medical Genetics and Genomics (4).

### Homology Modeling and Structural Analysis of *TK2* Missense Mutations

The structural model of human *TK2* enzyme was built based on the procedure described in previous report (5). Briefly, the Drosophila homolog deoxyribonucleoside kinase (PDB accession code: 1OT3) was used as template to construct the monomer structure of human *TK2* protein. The dimer structure is generated by a crystallographic symmetry through COOT software. The final *TK2* complex model is composed of amino acid residue 45–236, a thymidine molecule, and a sulfate molecule in each monomer.

### Muscle Biopsy

Twenty-three patients conducted muscle biopsy. Muscle samples were appropriately oriented and frozen in isopentane pre-cooled to −160°C in liquid nitrogen. Cryostat sections (8–10 μm) were cut from transversely oriented muscle blocks. Staining was performed with hematoxylin and eosin, modified Gomori trichrome (MGT), succinate dehydrogenase (SDH), cytochrome c oxidase (COX), oil red O (6).

### Statistical Analysis

The GraphPad Prism software (version 6.01) was used for statistical analysis. For variables distributed in a normal fashion, mean ± standard deviation was calculated. For non-normally distributed variables, medians were calculated. Student's *t*-test was performed for group pair. *P* < 0.05 was considered statistically significant.

## Results

### Patient Demographics

In our study, 24 suspected mitochondrial myopathy patients were enrolled, according to clinical presentation, lab findings, and pathological results by using muscle biopsy. Twenty-one patients reached genetic diagnosis (21/24, 87.5%, Table 1), consisting 10 girls and 11 boys. Four patients had a family history (case 2 and case 3 were siblings).

**Table 1:** Clinical and genetic characteristics of mitochondrial myopathy in children.

**No**	**Onset (y)**	**Gene**	**Variations**	**Mutation Load (%)**		**Muscular presentation**			**CNS presentation**			**Multisystem disease**				**Follow-up**
				**Proband**	**M relatives**	**OP**	**MW**	**EI**	**DR**	**seizure**	**ataxia**	**RF**	**HEART**	**GI**	**Other**	
1	0–3	MT-TL1	m.3243A>G	80.2(b)	91.4(mo)	+	+	+	+	–	–	+	+	+	–	Stable (BiPAP)
2	7–10	MT-TL1	m.3243A>G	ND	ND	–	+	+	–	+	–	+	–	–	–	Died at 11y.o.
3	0–3	MT-TL1	m.3243A>G	ND	ND	–	+	+	–	–	–	–	–	–	–	Slowly progressive
4	0–3	MT-TL1	m.3243A>G	85.6(b)	38.2(mo) 68.6(sis)	–	+	+	–	–	–	+	NA	NA	–	Died at 5y.o.
5	0–3	MT-TL1	m.3243A>G	91.4(m)	ND	–	+	–	+	–	–	+	–	NA	Swelling	Lost to follow-up
6	7–10	MT-TL1	m.3243A>G	97.1(m)	ND	+	+	–	–	–	–	+	+	+	Swelling	Stable (BiPAP)
7	0–3	MT-TL1	m.3243A>G	59.0 (b)	0(b) 0(sis)	–	+	+	–	+	–	+	+	+	Swelling	Stable (BiPAP)
8	7–10	MT-TL1	m.3243A>G	80.2(b)	ND	–	+	+	–	–	–	+	+	+	–	Tracheotomy and ventilation support
9	0–3	MT-TL1	m.3243A>G	95.0(b) 95.6(m)	ND	–	+	–	–	–	–	+	+	+	Hypoglycemia	Died
10	0–3	MT-TL1	m.3303C>T	97.2(m)	ND	–	+	+	–	–	–	–	+	+	–	Lost to follow-up
11	4–6	MT-TL1	m.3303C>T	36.0(b)	36.2(mo) 41.5(gra)	–	+	+	–	–	–	–	+	+	–	Relatively stable
12	7–10	MT-TL1	m.3302A>G	ND	ND	+	+	+	–	–	–	+	–	–	–	Died at 11y.o.
13	10–16	MT-TL1	m.3250T>C	99.6(m)	83.4(mo)	–	+	–	–	–	–	–	+	+	–	Stable
14	10–16	MT-TL1	m.3251A>G	64.6%(b)	ND	–	+	±	–	–	–	+	+	+	swelling	Died at 16y.o.
15	10–16	m.7702-14927del(b)		NA	ND	+	–	–	+	–	+	–	–	–	–	Stable
16	10–16	m.8502-13377del(m)		NA	ND	+	–	+	–	–	–	–	–	–	–	Stable
17	7–10	m.8380-13600del(m)		NA	ND	+	–	–	–	–	+	–	+	–	PDR	Stable
**No**	**Onset(y)**	**Gene**	**Variations**		**Interpretation (ACMG criteria)**	**Muscular presentation**			**CNS presentation**			**Multisystem disease**				**Follow-up**
						**OP**	**MW**	**EI**	**DR**	**seizure**	**ataxia**	**RF**	**HEART**	**GI**	**Other**	
18	0–3	TK2	c.144_145del(p.Glu48fs)		pathogenic	–	+	–	–	–	–	+	NA		–	Died before 3 y.o.
			c.C547T(p.Arg183Trp)		pathogenic											
19	0–3	TK2	c.T659C(p.Leu220Pro)		Likely pathogenic	–	+	–	–	–	–	+	–		Swelling	Died at 2 y.o.
			c.A497T(p.Asp166Val)		Likely pathogenic											
20	0–3	TK2	c.497A>T(p.Asp166Val)		Likely pathogenic	–	+	–	–	–	–	+	–	–	–	Died at 3 y.o.
			c.328C>T(p.Gln110*)		pathogenic											
21	0–3	SURF1	c.752_755del(p.Gln251Profs*15)		pathogenic	–	+	–	–	–	–	–	–	–	–	Lost to follow-up
			c.769G>A (p.Gly257Arg)		pathogenic											

*M relative, maternal relative; OP, Ophthalmoplegia; MW, muscle weakness; EI, exercise intolerance; DR, developmental delay; RF, respiratory failure; GI, gastrointestinal involvement; mo, mother; sis, sister; gra, maternal grandmother; MDR, mental developmental retardation; b, blood; m, muscle; y.o., years old; Nor, normal; ND, not done; NA, not available/applicable; PDR, pigmentary degeneration of retina*.

### Clinical Presentation

All 21 genetic diagnosed MM patients were term healthy babies after uneventful deliveries with normal birthweight. On developmental history, all patients met their milestones before the onset of disease except two cases had slightly delayed first steps (2/21, 9.5%). However, among these patients who once were able to walk, retrospection revealed that most of them were less athletic compared to their peers in childhood (10/17, 58.8%).

The median onset age was 3.0 years old (ranging from 1 day after birth to 15 years old), and the most common onset presentation was limb muscle weakness or fatigue (13/21, 61.9%), followed by sudden respiratory failure demanding of intubation and ventilator (3/21, 14.3%), ptosis (2/21, 9.5%), myalgia with muscle weakness and swelling (2/21, 9.5%), tremor of hands who developed ptosis after 2 years (1/21, 4.8%). For the initial clinical diagnosis, the most common was congenital myopathy (5/21, 23.8%), followed by myopathy (3/21, 14.3%), metabolic myopathy (3/21, 14.3%), myasthenia gravis (3/21, 14.3%), respiratory failure (2/21, 9.5%), hereditary metabolic disease (2/21, 9.5%), myositis (1/21, 4.8%), muscular dystrophy (1/21, 4.8%), and myocarditis (1/21, 8.4%). The median duration of genetic diagnosis from initial onset was 6 months (ranging from 15 days to 12 years).

During the disease course, 18 patients developed proximal muscle weakness (18/21, 85.7%), 7 patients had axial muscles especially neck extensor muscle weakness (7/21, 33.3%), and 6 cases had ptosis (6/21, 28.6%). Three patients developed torticollis (3/21, 14.3%), 3 had winged scapula (3/21, 14.3%), 3 had scoliosis with or without chest deformity (3/21, 14.3%) (Supplementary Table 1).

Blood test demonstrated mild to severe elevated creatine kinase (CK) levels (median of 908.5 IL/L, ranging from 30 to 8,000 IU/L, Normal range: 0–164 IU/L) in 15 patients (15/21, 71.4%) and elevated lac acid (median of 5.35 mmol/L, ranging from 1.5 to 14.3 mmol/L, Normal range: 0–2.1 mmol/L) in 17 patients (17/19, 89.5%). There is fluctuating of CK and lactate acid level during the disease course of mitochondrial myopathy: relatively low at stable situation while higher during metabolic crisis. Blood and urine tandem mass spectrometry conducted in some patients revealed that two patients showed elevated alanine and glycine (2/5, 40%), respectively, whilst six patients showed abnormal organic acids (6/7, 85.7%). Eleven patients showed myogenic damage in EMG tests (11/14, 78.6%), while one patient presented both myogenic and neuropathic finding (1/14, 7.1%) (Supplementary Table 2).

Multisystemic symptoms were observed (Table 1 and Supplementary Material). Five patients presented with CNS symptoms including seizure, mental regression, ataxia (5/21, 23.8%), while cranial MRI demonstrated abnormal findings in 10 patients (10/17, 58.8%), and the lesion mainly consist of basal ganglia, brain stem, cerebellum, and deep white matter. Ten patients revealed heart disease: echocardiogram showed 5 cases had abnormal change (5/15, 33.3%) among which the most common findings were cardiac enlargement, tricuspid regurgitation, and pulmonary artery hypertension; electrocardiogram study was available in 13 patients which demonstrated abnormal results for 6 cases (6/13, 46.2%) including sinus tachycardia, high voltage, and atrioventricular block. Five patients had complaints at least once for gastrointestinal problems, mostly referring to recurrent vomiting and abdominal distension (5/21, 23.8%). And five patients had experienced swelling of hands, arms, and feet in the duration of disorder (5/21, 23.8%), with cardiac and hepatogenic cause ruled out.

**Figure 1:**
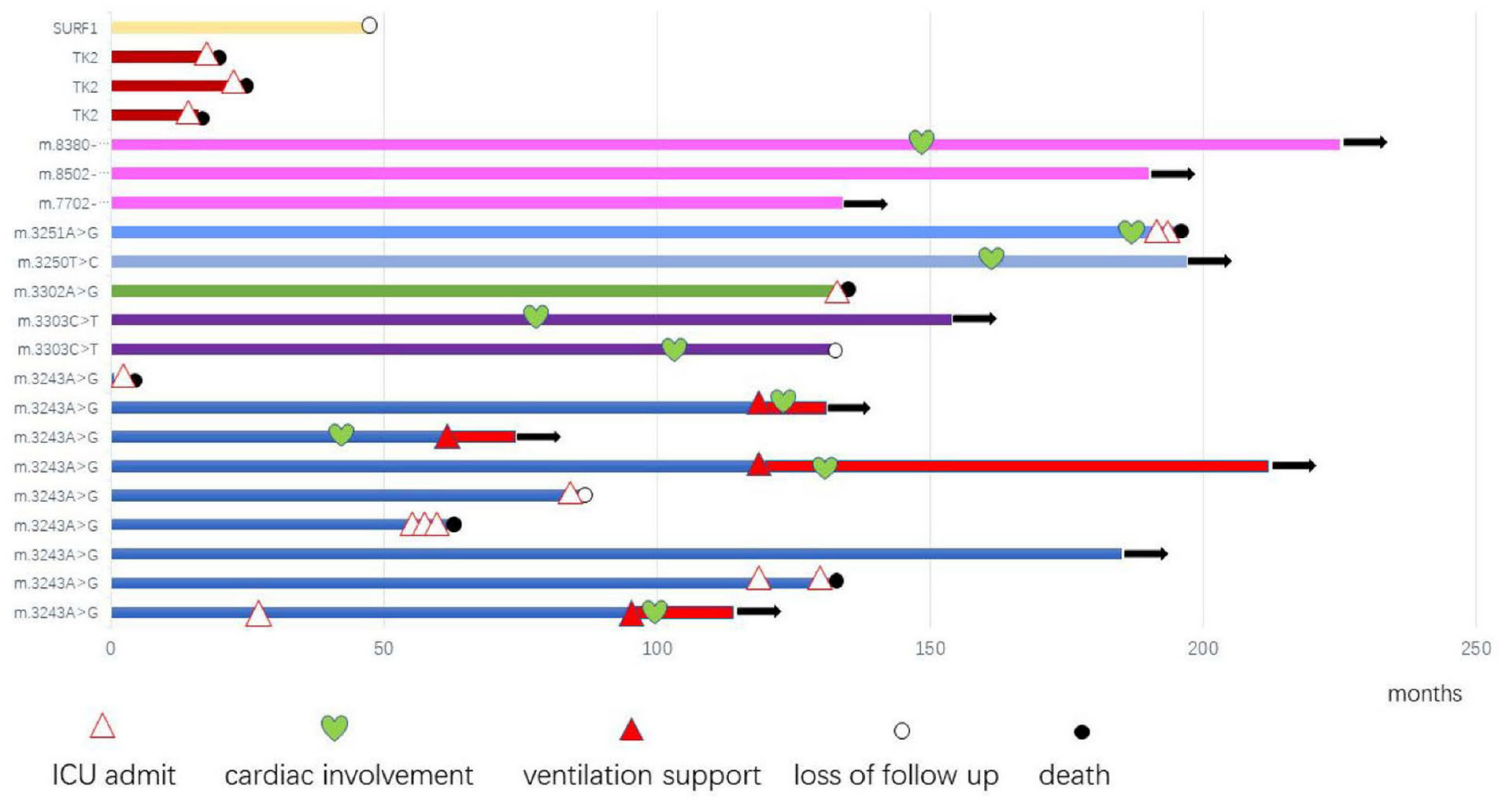
The profile and outcome of 21 genetic diagnosed mitochondrial myopathy patients.

After diagnosis, all patients received Cock-tail treatment including multi vitamins, cofactors, and nutritional supplements such as CoQ10 and L-carnitine. Eighteen patients were followed up regularly (18/21, 85.7%), up to 7 years (median of 1 years, range 15 days−7 years). Thirteen patients had been admitted to the intensive care unit (ICU) because of hypercapnic respiratory (13/21, 61.9%), 8 of them died from recurrent or refractory respiratory failure (8/21, 38.1%), 4 patients stay relatively stable with BiPAP, 1 patient was lost to follow up after his parents gave up, and 1 patient received tracheotomy and persistent ventilation support (Figure 1).

### Molecular and Genetic Results

Twenty-one patients had a genetic diagnosis (21/24, 87.5%), including 14 patients with 6 mtDNA point mutations (14/21, 66.7%), 3 patients with large scale mtDNA deletions (3/21, 14.3%), and 4 patients with 7 nuclear gene mutations (4/21, 19.0%).

Fourteen MM patients were found to harbor 6 reported mtDNA point mutations, including m.3243A>G (9/15, 60%) (7, 8), m.3303C>T(2/15, 13.3%) (9), m.3302A>G(1/15, 6.7%) (10), m.3250T>C(1/15, 6.7%) (11), and m.3251A>G(1/15, 6.7%) (12, 13). The mutation load of m.3243A>G variation ranged 59.0–95.0% in blood while 91.4–97.1% in muscle tissue, among maternal family members who received mtDNA test as well 3 maternal relatives with higher heteroplasmy level however demonstrated no obvious symptoms (Table 1, case 1, and case 11).

Three patients revealed novel single large deletions, including m.7702-14927del from blood, m.8502-13377del, and m.8380-13600del from muscle tissue, all of which were considered to be pathogenic (14).

In our cohort, 5 mutations in *TK2* gene were identified in 3 patients (3/21, 14.3%) by nuclear gene panel, including one common reported c.547C>T (p.R183W) mutation (5) and 4 novel variants: c.659T>C (p.L220P), c.497A>T (p.D166V), c.328C>T (p.Q110^*^), and c.144_145delTC, which had been interpreted as pathogenic or likely pathogenic according to the ACMG criteria (4) and have been submitted to the public database ClinVar (http://www.ncbi.nlm.nih.gov/clinvar, submission number: SUB6664717). Furthermore, analysis of mitochondrial DNA content in muscle tissue of these patients have confirmed remarkable reduction compared to healthy controls (11.30–13.25%) in our previous report (15). One patient revealed mutations in *SURF1* gene, including one missense mutation of c.769G>A(p.Gly257Arg) which had been reported to cause Leigh syndrome (16) and one novel frameshift mutation of c.752_755del(p.Gln251Profs^*^15).

### Homology Modeling and Structural Analysis

**Figure 2:**
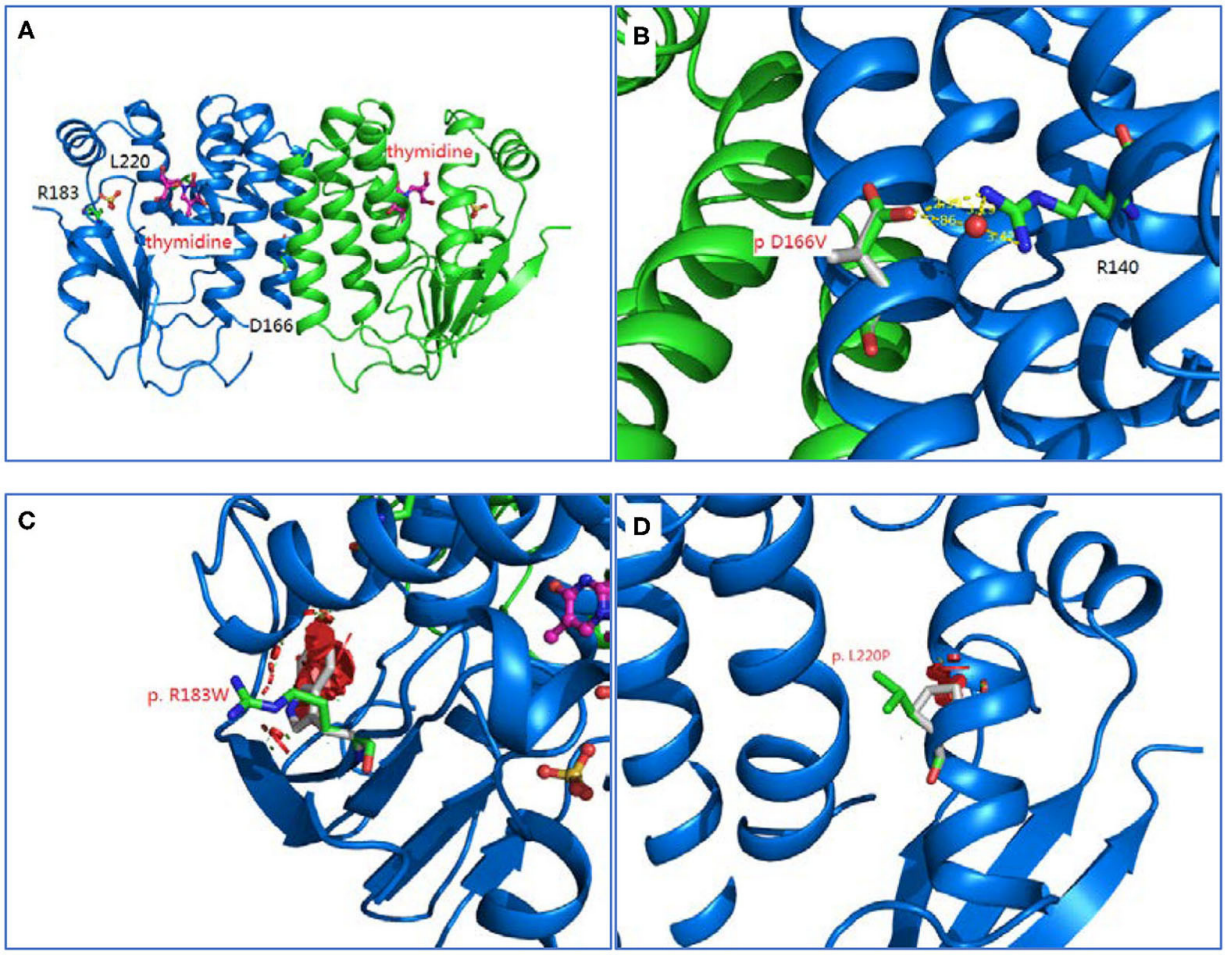
Homology modeling of 3 missense variants of *TK2* gene. **(A)**: Legend: Ribbon diagram of complex structure model of human *TK2* dimer. **(B)**: Asp166 side chain is an integral part of close interaction with Arg140. Replacement of Asp with Val disrupts the stereochemical interactions and introduces unfavorable. **(C)**: Arg183 is located on the surface of *TK2* enzyme, and interacts with solvent extensively. Replacement of hydrophilic Arg with Trp introduces the hydrophobic residue to the surface of protein and creates severe steric clashes with adjacent residues. **(D)**: Leu220 is located at the middle of helical structure, and maintain this configuration through proper peptide bond. Replacement of Leu with Pro disrupts the proper peptide bond for this helix and creates steric clashes with adjacent residues.

Homology modeling of missense variants of *TK2* gene including c.547C>T (p.R183W), c.659T>C (p.L220P), and c.497A>T (p.D166V) were displayed (Figure 2).

### Morphological and Histopathological Analyses of the Muscle Samples

Among the 21 genetic diagnosed patients, 20 patients received muscle biopsy. Seventeen patients showed remarkable variation in fiber size (17/20, 85%) in HE staining, fragmented muscle fibers were observed in 19 patients with alkaline particles in irregular cellular or sub-sarcolemma fissure (19/20, 95%), however, degeneration, necrosis, and regeneration in fibers were rarely observed (2/20,10%). In GMT staining, Ragged-red fibers (RRFs) were found in 17 patients (17/20, 85%) with the proportion of RRFs ranging from 4.5 to 47.3%, while 17 patients revealed COX-negative fibers (17/20, 85.0%) with the proportion ranging from 0.01 to 73.3%, and 12 patients presented with strongly succinate dehydrogenase-reactive blood vessels (SSV) phenomenon (12/20, 60.0%) (Supplementary Material). Seven patients had mild glycogen storage (7/20, 35%) and 10 cases showed mild lipid storage in muscle fibers (10/20, 50%).

### Correlation Between Phenotype and Genotype

**Figure 3:**
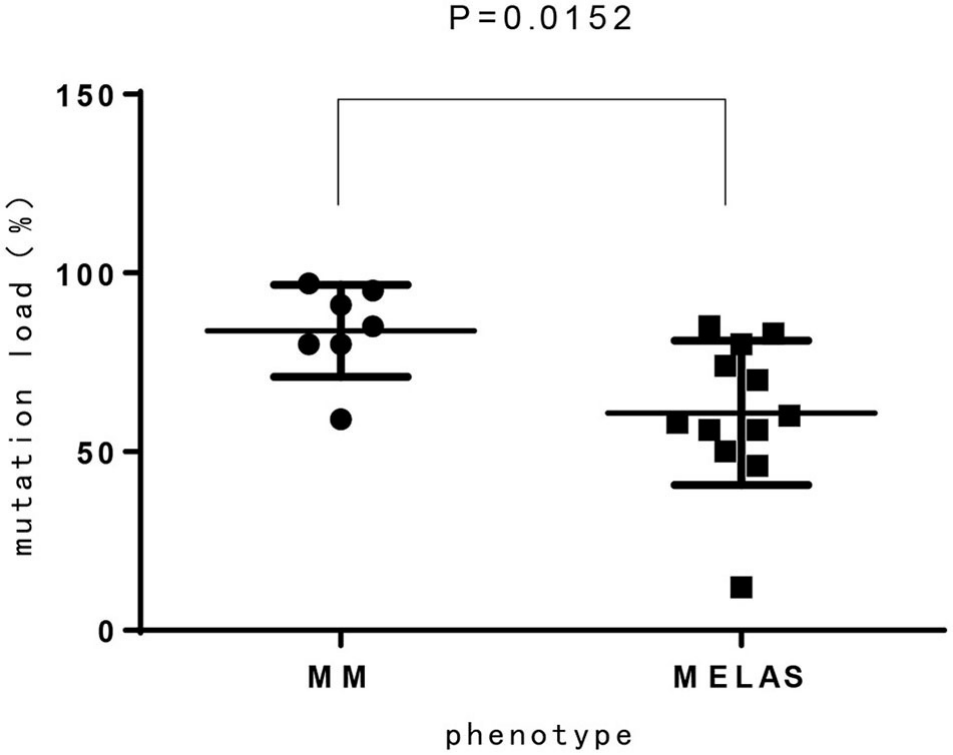
The mutation load of m.3243A>G between MM and MELAS patients.

Among 14 patients with mtDNA point mutation, most cases presented with progressive proximal mitochondrial myopathy (12/14, 85.7%), including patients with m.3243A>G mutation(*n* = 8), m.3303C>T mutation(*n* = 2), and m.3250T>C, m.3251A>G for one case, respectively. One of the exceptions is a neonate who harboring heteroplasmy level of m.3243A>G presented with hypoglycemia, respiratory failure, refractory hyperlactacidemia as well as pathologically demonstrated mitochondrial myopathy. Another patient who ga ined m.3302A>G variant showed clinical characteristic consistent with CPEO. Compared to MELAS patients with m.3243A>G mutation from our previous report (15), the mutation load of m.3243A>G in MM is higher than MELAS, which has significant difference (Figure 3).

Three patients were found to harbor single large-scale deletion of mtDNA in our study, including one patient with m.8380-13600 deletion presenting with KSS whose parents declined refractory sideroblastic anemia during her infancy as previous report (14), and two patients with m.7702-14927 deletion and m.8502-13377 deletion who showed symptoms of CPEO.

Compared to the high clinical heterogeneity of mtDNA mutated MMs, patients with *TK2* mutation presented consistently with early onset of muscle weakness, rapid deterioration, and failure to survive during early childhood. Additionally, remarkably higher proportion of RRFs and COX-negative fiber were found in *TK2* mutated patients.

One patient with *SURF1* gene mutations in our study presented with progressive myopathy and characteristic pathological finding in muscle: massive COX negative fibers without any RRFs.

## Discussion

Skeletal muscle is commonly affected in mitochondrial disease because of high energy demand (11, 12, 16, 17), but erroneous and delaying diagnosis of mitochondrial myopathy is not rare due to clinical and genetic heterogeneity as well as non-specific presentation in some patients (17). In our study, 47.6% of MM patients were misdiagnosed initially to be congenital myopathy, respiratory failure, or myasthenia gravis, etc.

In our study, 16 cases presented with progressive proximal mitochondrial myopathy (16/21, 76.2%), including 9 patients with m.3243A>G mutation, which commonly cause mitochondrial encephalomyopathy, lactic acidosis, and stroke like episodes (MELAS), maternally inherited diabetes and deafness (MIDD), or CPEO (18). Additionally, from our investigation, the mutation load of m.3243A>G in MM is higher than that of MELAS, which has significant difference (*p* < 0.05). It is likely demonstrating the correlation between heteroplasmy level and the phenotype. We also observed that three maternal relatives (case 1 and case 11) harboring higher mutation load in blood however presented no symptom or abnormal lab findings, nevertheless the mutation load in muscle tissue is not available. There was once a report that found 9% of individuals harboring m.3243A>G mutation were clinically asymptomatic (18), thus, in our opinion heteroplasmy is not yet the whole story about the phenotypic variability, the tissue heterogeneity, heritable nuclear factor or some other unknown modifying mechanism is likely playing a role and to be revealed.

In present study, mtDNA mutations are more common than nuclear gene defects (17:4), of which mitochondrial tRNAs mutations are most prevalent, which is consistent with previous report (7). However, with the development of next generation sequencing, more nuclear gene defects are being found to cause MM (19). In present report, three patients remained without definite genetic diagnosis despite whole mtDNA and nDNA sequencing (14.3%). This may be because that the variant being detected is not prioritized by current bioinformatic pipelines or may be because the causative variant does not reside in the coding regions of the genome (19). It is hopeful that with the further development of novel molecular genetic strategies such as whole genome sequencing and RNA-seq novel disease genes are going to be identified.

Despite of the clinical and genetic heterogeneity, there is correlation between the two of them. Patients with m.3303C>T, m.3250 T>C, m.3251A>G and m.3243A>G mutation are in relatively higher risk to develop cardiomyopathy, while patients with m.3243A>G mutation and large-scale deletion are more likely to develop CNS presentation or cranial lesion as previously reported (14). *TK2* mutated MM patients always onset during late infancy with progressive motor regression and failed to survive at early childhood. Different patterns of pathological finding in muscle biopsy is observed among MMs with different gene defects: myopathic damage with sporadic RRFs and COX negative fibers, sometimes to high extent, is the feature of patients with mtDNA point mutations such as m.3243A>G^1^, profound RRFs and COX negative fibers was common in *TK2* mutated patients, while overall COX negative fibers without RRF was found in *SURF1* mutated patient, which is consistent with the complex IV assembly function.

From the follow-ups, MM patients with m.3243A>G, m.3302A>G and m.3251A>G mutations possibly tends to have poor prognosis due to early respiratory failure. Respiratory failure is a common and life-threatening condition in metabolic myopathy that demands prompt diagnosis, assessment and appropriate management (20). A report referring to 22 hereditary myopathies with early respiratory insufficiency in adults pointed out that a MELAS patient with m.3243A>G mutation suffered from orthopnea and dyspnea on exertion at 39 years old (21). Another report studied adults with apnea, and implied that 8.5% patients were finally diagnosed as mitochondrial myopathy (17). There were only few case reports about acute respiratory failure (22) as well as death due to respiratory failure in pediatric mitochondrial myopathy patients (7, 12, 23, 24). Based on our study, respiratory failure with hypercapnia was probably the main cause of death in pediatric mitochondrial myopathy patients, especially those with m.3243A>G, m.3302A>G, and m.3251A>G mutations. That is possibly because respiratory muscle weakness, chest wall, and spinal deformities in MMs lead to an inability to maintain a level of minute ventilation appropriate for the rate of carbon dioxide production resulting hypercapnia, with or without concomitant hypoxemia. Another explanation that significant mortality occurs in these patients is that they have chronic respiratory disorder and other comorbidities such as cardiopulmonary, or neurologic disease, as well as poor nutritional status.

To date, there are no effective treatments to halt the progression of MM, and therapeutic options still focus on the symptomatic management of disease, mainly including restorative (such as Q10 supplementation in primary genetic defects of coenzyme Q10 synthesis) or preventative strategies during episodes of acute metabolic decompensation due to physiological stressors (such as dehydration, fever, surgery, sepsis) (25), which may improve quality of life and potentially increase life expectancy. From our study, the potential respiratory insufficiency resulting from respiratory muscle weakness and skeletal deformities is easy to be underrated, till acute hypercapnic respiratory failure requiring intubation and mechanical ventilation occur. Although there are more than 50 clinical trials currently targeting primary mitochondrial diseases, the evidence for most pharmacological strategies still remains controversial (26). A double-blind, randomized, placebo-controlled, cross-over trial of resveratrol (an activator of AMPK and SIRT1) in patients with mitochondrial myopathy has completed recently but the results have not been published. Furthermore, new small molecular and cellular strategies such as mtZFNs and mtTALENs have shown a beneficial shift in heteroplasmy and improvement in the biochemical deficit of MD (1, 27). It is hopeful that major breakthroughs will occur during the next decade in the treatments for mitochondrial disease.

## Conclusion

Mitochondrial myopathy in children has great clinical, pathological, and genetical heterogeneity. Progressive proximal myopathy is most prevalent, while mtDNA point mutations are most common. Multi-system involvement is common and respiratory failure is a critical risk factor of mortality. New target therapy of mitochondrial myopathy needs to be studied furtherly.

## Data Availability Statement

The raw data supporting the conclusions of this article will be made available by the authors, without undue reservation.

## Ethics Statement

The studies involving human participants were reviewed and approved by The health authority ethical committee of Children's Hospital of Fudan University. Written informed consent to participate in this study was provided by the participants' legal guardian/next of kin.

## Author Contributions

CH prepared and drafted this manuscript. YS conducted the electromyogram tests. SZ is responsible for the clinical genetic diagnosis. LZ took charge of the pathological analysis. YW and XL fulfill data analysis and approve for the submission. All authors contributed to the article and approved the submitted version.

## Conflict of Interest

The authors declare that the research was conducted in the absence of any commercial or financial relationships that could be construed as a potential conflict of interest.
