# Supplementary material for: Clinical Profile and Outcome of Pediatric Mitochondrial Myopathy in China
Source: Front Neurol. 2020 Sep 8;11:1000. doi: 10.3389/fneur.2020.01000 (PMC7506116; doi:10.3389/fneur.2020.01000)
Supplement: Supplementary file 1 [file Table_1.DOCX]

Table 1 Muscle weakness and skeletal deformity of 21 genetic diagnosed mitochondrial myopathy patients

| No | EI | Ptosis | PUL | DUL | PLL | DLL | Neck flexor | deformity |
| --- | --- | --- | --- | --- | --- | --- | --- | --- |
| 1 | + | + | 1 | 0 | 2 | 0 | 3 | torticollis |
| 2 | + | - | 1 | 0 | 1 | 0 | 3 | / |
| 3 | + | - | 1 | 0 | 1 | 0 | 2 | torticollis; scoliosis; lumbar lordosis; chest deformity; winged scapular |
| 4 | + | - | 2 | 1 | 2 | 1 | 3 | torticollis; lumbar lordosis; chest deformity; scoliosis |
| 5 | - | - | 2 | 0 | 2 | 0 | 2 | - |
| 6 | - | + | 2 | 0 | 2 | 0 | 3 | chest deformity |
| 7 | + | - | 2 | 1 | 2 | 1 | 3 | - |
| 8 | + | - | 1 | 0 | 1 | 0 | 1 | Scoliosis |
| 9 | - | - | 1 | 1 | 1 | 1 | 1 | - |
| 10 | + | - | 1 | 0 | 1 | 0 | 2 | winged scapular; Gower sign |
| 11 | + | - | 1 | 0 | 2 | 0 | 1 | - |
| 12 | + | + | 2 | 1 | 2 | 0 | 3 | winged scapular |
| 13 | - | - | 1 | 0 | 1 | 0 | 1 | - |
| 14 | +/- | - | 3 | 2 | 3 | 2 | 3 | contracture of knee joint |
| 15 | - | + | 0 | 0 | 0 | 0 | 0 | - |
| 16 | + | + | 0 | 0 | 0 | 0 | 0 | - |
| 17 | - | + | 0 | 0 | 0 | 0 | 0 | - |
| 18 | - | - | 3 | 2 | 3 | 2 | 3 | - |
| 19 | - | - | 3 | 2 | 3 | 2 | 3 | - |
| 20 | - | - | 3 | 2 | 3 | 2 | 3 | - |
| 21 | - | - | 2 | 0 | 2 | 0 | 2 | abnormal gait |

EI: exercise intolerance; PUL: weakness of proximal upper limb; DUL: weakness of distal upper limb; PLL: weakness of proximal lower limb; DLL: weakness of distal lower limb; Strength: 0: normal; 1: mild weakness (Grade 4-5); 2: moderate weakness (Grade 3-4); 3: severe weakness (< Grade 3)
