# Supplementary material for: Clinical Profile and Outcome of Pediatric Mitochondrial Myopathy in China
Source: Front Neurol. 2020 Sep 8;11:1000. doi: 10.3389/fneur.2020.01000 (PMC7506116; doi:10.3389/fneur.2020.01000)
Supplement: Supplementary file 2 [file Table_2.DOCX]

The laboratory and pathological results of 21 genetic confirmed MM patients

| Case | CKmax  (25-200IU/L) | LACmax  (<2.1mmol/L) | Cranial MRI | EMG | ECHO & EKG | MM | Muscle Pathology | |
| --- | --- | --- | --- | --- | --- | --- | --- | --- |
|  |  |  |  |  |  |  | RRF | COX-fibers |
| 1 | 4712 | 8.2 | Nor | Nor | Mild tricuspid regurgitation | NA | + | + |
| 2 | 112 | 2.3 | Cortex lesion | Myopathic | Nor | NA | + | + |
| 3 | 273 | 5.1 | NA | Myopathic | Nor | NA | + | + |
| 4 | 426 | 6.5 | Nonspecific lesion | Myopathic | NA | NA | + | - |
| 5 | 8000 | NA | NA | NA | NA | NA | + | ++ |
| 6 | 690 | 5.6 | Nor | Myopathic | Pulmonary artery hypertension  Tricuspid regurgitation  pericardial effusion  enlargement of Right atrium and ventricle  ST-segment change | NA | ++ | +++ |
| 7 | 623 | 9.6 | basal ganglia | Myopathic | Enlargement of left ventricle  mild mitral regurgitation | 4-hydroxyphenylacetic acid↑  aconitic acid↑ lactic acid↑ | + | + |
| 8 | 197 | 14.5 | bilateral basal ganglia  white matter  unilateral thalamus | NA | Right axis deviation | Citric acid↑  aconitic acid↑↑ | - | - |
| 9 | 972 | 14.3 | NA | NA | Thickening of ventricular septum and left ventricular posterior wall  patent ductus arteriosus  patent foramen ovale  pulmonary artery hypertension  Tricuspid regurgitation | Lactic acid↑↑↑ pyruvic acid↑↑↑  2-oxoglutaric acid ↑↑↑  3-hydroxyisobutyric acid↑↑  2-hydroxyisovalerate↑↑  boletic acid↑ 2-ketohexanoic acid ↑ | - | +++ |
| 10 | 845 | NA | Nor | Myopathic | High P wave  high voltage of Left ventricle | NA | + | + |
| 11 | 3199 | 10.2 | Nor | Myopathic | sinus tachycardia and T wave change in partial period  premature beat in atrioventricular junction | Lactic acid↑hydroxybutyric acid↑pyruvic acid↑ | + | + |
| 12 | 381 | 10.4 | nonspecific | Myopathic | Nor | NA | ++ | + |
| 13 | 7057 | 13.0 | NA | Myopathic | Normal ECHO; sinus tachycardia | NA | + | - |
| 14 | 4231 | 10.2 | Nor | NA | Generalized cardiac enlargement | 3-hydroxybutyric acid↑↑  Lactic acid↑↑ | ++ | +++ |
| 15 | 101 | 4.3 | cerebelli brain stem  basal ganglia  subcortical white matter | Myopathic and neuropathic | Nor | Citric acid↑↑ | NA | NA |
| 16 | 107 | 4.6 | basal ganglia | Myopathic | Nor | NA | + | + |
| 17 | 130 | 1.5 | minor deep white matter | NA | I° atrioventricular heart-block | NA | + | + |
| 18 | 2270 | 2.1 | thin callosum | Myopathic | NA | NA | ++ | ++ |
| 19 | 1465 | 4.8 | Nor | Nor | Nor | NA | ++ | ++ |
| 20 | 7276 | 5.0 | Nor | NA | Nor | Nor | ++ | ++ |
| 21 | 94 | 4.9 | basal ganglia | NA | Nor | NA | - | ++++ |

CK: creatine kinase; Lac: serum lactate acid; EMG: electromyogram; ECHO: echocardiogram; EKG: electrocardiogram; RRF: ragged-red fiber; COX: cytochrome oxidase; RRF: ragged-red fiber; COX: cytochrome oxidase; - negative; + <25%, ++ 25%~50%, +++ 50%~75%，++++ >75%; Nor: normal; NA: not available
